# Cytochrome P450 as possible link between Mallory-Denk body formation in DDC-intoxicated mouse liver and human steatohepatitis

**DOI:** 10.1007/s00428-025-04290-4

**Published:** 2025-10-30

**Authors:** Cornelia Stumptner, Karoline Fechter, Kurt Zatloukal, Helmut Denk

**Affiliations:** Diagnostic and Research Institute of Pathology, Center of Molecular Biomedicine, Medical University of Graz, Neue Stiftingtalstrasse 6, 8010 Graz, Austria

**Keywords:** Alcohol-related steatohepatitis (ASH), Metabolic dysfunction-associated steatohepatitis (MASH), Reactive oxygen species (ROS), CYP2A5, Bilirubin, Oxidative stress

## Abstract

Mallory-Denk bodies (MDBs) are hepatocytic inclusions in alcohol-related (ASH) and metabolic dysfunction-associated (MASH) steatohepatitis as well as in some other apparently etiologically different chronic human liver disorders. They are experimentally induced in mouse liver by chronic intoxication with 3,5-diethoxycarbonyl-1,4-dihydrocollidine (DDC). Like in human diseases, mouse liver MDBs consist of stress proteins as major components indicating their relationship to chronic cellular stress. Mouse livers at different stages of DDC intoxication, recovery, and re-intoxication were analyzed with regard to cytochrome P450 (CYP) expression, enzyme activity, oxidative stress, and anti-oxidative response. mRNAs of cytochrome P450 2A5 (CYP2A5), heme oxygenase-1 (Hmox-1), and the ubiquitin-binding adapter protein p62/sequestosome1 (p62) were significantly elevated by DDC treatment. CYP2A5 protein correlated at the cellular level with the presence of MDBs, and CYP2A5-related coumarin 7-hydroxylase (COH) activity was increased in relation to MDB expression. A functional relationship between CYP2A5 and Hmox-1 activity was established by the identification of bilirubin as a potent inhibitor of the production of reactive oxygen species (ROS) by CYP2A5. Moreover, CYP2E1 as well as CYP2A6, which are induced in human steatohepatitis, showed similar H_2_O_2_ production and inhibition by bilirubin in vitro. These findings suggest similar pathogenic mechanisms involved in MDB formation related to the overexpression of ROS-producing CYP2A5 in mice and CYP2E1 and CYP2A6 in human steatohepatitis.

**Supplementary Information:**

The online version contains supplementary material available at 10.1007/s00428-025-04290-4.

## Introduction

Mallory-Denk bodies (MDBs) are cytoplasmic inclusions in hepatocytes associated with a variety of chronic liver disorders, particularly alcohol-related (ASH) and metabolic dysfunction-associated (MASH) steatohepatitis [1]. Moreover, several other etiologically different chronic liver diseases, such as chronic cholangiopathies, copper toxicosis, hepatocellular carcinomas, focal nodular hyperplasia and liver injuries induced by drugs and chemicals may lead to MDB formation [1, 2]. MDBs are aggregates with filamentous ultrastructure composed of abnormally folded, hyperphosphorylated, partially degraded, cross-linked and ubiquitinated keratins, heat shock proteins and the stress-induced, ubiquitin-binding adapter protein p62/sequestosome1 (p62) as major constituents [1, 2]. Morphologically and chemically identical inclusions are induced in mouse hepatocytes by chronic intoxication with the porphyrinogenic agents griseofulvin or 3,5-diethoxycarbonyl-1,4-dihydrocollidine (DDC), in livers of aged keratin 18 (K18) knock-out mice, in transgenic mice overexpressing human K8, in ferrochelatase-deficient porphyric mice, and in tissue culture cells after transfection and overexpression of MDB components [1–10].

A notorious difficulty inherent in the evaluation of chronic diseases, particularly clinically relevant interpretation of morphologic features, is that these may be the result of a complex interplay between the damaging effects of noxious insults and adaptative cellular responses. Despite the detailed molecular characterization of MDBs, the biological, but also clinical, significance of MDBs is still debated. In this context the question arises whether they are the result of direct cell damage or of cellular adaptation to chronic stress. Because MDBs are essentially identical in humans and mice, it can be expected that understanding the mechanisms of MDB formation in the experimental model provides insight into pathogenic principles involved in steatohepatitis and other MDB-associated liver diseases of humans.

The experimental induction of MDBs in mice requires long-term (chronic) intoxication with DDC; however, after recovery followed by the disappearance of most MDBs, they are rapidly re-induced by short-term re-exposure allowing to discriminate effects of chronic intoxication from those more acutely related to MDB formation [1, 2, 4–7]. The mechanisms underlying this “toxic memory” effect is unclear. In this study we tested the hypothesis whether induction of cytochrome P450 (CYP) and related oxidative stress could be involved. DDC is metabolized by CYPs, particularly CYP2A5, acts as heme methylating agent and leads to the accumulation of the potent ferrochelatase inhibitor N-methyl protoporphyrin IX (PP-IX) in the liver [11]. Porphyrins cause cellular energy imbalance by aggregating key glycolytic enzymes and producing mitochondrial dysfunction [10, 11]. Moreover, it has been demonstrated in studies of genetic and inducible models of porphyria that PP-IX caused keratin (K) 8/18 and nuclear lamin (A/C/B1) aggregation [11]. Since CYPs, particularly CYP2E1, play an important role in ASH and MASH (12–17) the role of CYPs, related oxidative stress, and antioxidative cellular responses appeared to be particularly relevant in this context.

## Materials and methods

### Chemicals and commercial CYP preparations to be used in the in vitro studies

Unless stated otherwise, chemicals were from Sigma-Aldrich (Vienna, Austria), Merck (Vienna, Austria) or Fluka (Buchs, Switzerland) and were at least of analytical grade. CYP2E1 and CYP2A6 supersomes, containing the respective CYP and cytochrome b5, were obtained from BD Biosciences (Heidelberg, Germany). Lucigenin was purchased from Invitrogen (Lofer, Austria).

### Animal experiments

Three to six male Swiss Albino mice (strain Him OF1 SPF; Institute of Laboratory Animal Research, Medical University of Vienna, Himberg, Austria) were studied in each experimental setting. They were fed either a standard diet (Ssniff Spezialdiäten GmbH, Soest, Germany) for control purposes or the diet containing 0.1% DDC (Sigma-Aldrich, Vienna, Austria) for 3 days (acute intoxication regimen) or for 2.5 months (chronic intoxication regimen). After 2.5 months of continuous DDC feeding (“priming”) a group of animals was allowed to recover on standard diet for 1 month. Recovered mice were then re-intoxicated with DDC for 3 days (“trigger”) for re-induction of MDBs. The animals were kept at specific pathogen-free conditions in individually ventilated cages (Bio.A.S. ventilation unit, Ehret Labor- und Pharmatechnik GmbH & Co. KG, Emmendingen, Germany) on a 12 h light/dark cycle. Mice were sacrificed by decapitation between 1 and 2 pm, since circadian rhythms influence CYP expression, and blood was collected for further analysis. Serum samples were analyzed with a Hitachi 717 analyzer (Boehringer Mannheim, Germany). Liver tissue was snap-frozen in methylbutane pre-cooled with liquid nitrogen for RNA isolation, immunofluorescence analysis and enzyme activity assays or fixed in 3.7% neutral formaldehyde solution and embedded in paraffin for routine hematoxylin–eosin (H&E) staining. The animal experiments were approved by the Austrian Federal Ministry of Education, Science and Research. The mice received humane care according to the criteria outlined in the Guide for the Care and Use of Laboratory Animals (prepared by the National Academy of Sciences and published by the National Institute of Health; NIH publication 86–23, revised 1985).

### Quantitative reverse transcriptase polymerase chain reaction (PCR) for mRNA determination

Total RNA was isolated from liver tissue using TriReagent (Molecular Research Center, Inc., Cincinnati, OH), DNAse I digested, cleaned up using the NucleoSpin RNA II (GenXpress, Wiener Neudorf, Austria) and reverse-transcribed into complementary DNA by using the Superscript II Reverse Transcriptase (Invitrogen, Vienna, Austria) according to the instructions of the supplier. PCR was performed in a 30 µl reaction mixture using either TaqMan Universal PCR Master Mix (Applied Biosystems, Waltham, MA, USA) or SYBRgreen PCR Master Mix (Applied Biosystems). Primers (synthesized by MWG-Biotech AG, Ebersberg, Germany) are listed in Table 1. Samples were analyzed in duplicates using the ABI PRISM 7900 Sequence Detection System (Applied Biosystems). Standard curves were generated with 50, 25, 12.5, 5 and 1 ng cDNA. 28 s RNA served as reference.

**Table 1:** Primers

Gene	Acc.no	Primer/probe (dye label)	Sequence 5’ – 3’	
CYP2A5	NM_007812	Forward	CCTTAGCCGAACAGTCTCCAATG	124 bp
		Reverse	AACATCTCATAGAGCTGCCCCAT	
		Probe	GCTTTGACTATGAGGACAAAGAGTTCCTGTCAC	
		(FAM/TAMRA)		
Hmox-1	NM_010442	Forward	CACTTCGTCAGAGGCCTGCTA	93 bp
		Reverse	GTCTGGGATGAGCTAGTGCTGAT	
p62	NM_011018	Forward	ACCCACAGGGCTGAAGGAAGCT	124 bp
		Reverse	TGGTGAGCCAGCCGCCTTCAT	
		Probe (FAM/TAMRA)	CGGCTGATTGAGTCCCTCTCCCAGATGCTGTCC	
28 s RNA	X00525	Forward	CGGCTCTTCCTATCATTGTG	97 bp
		Reverse	CCTGTCTCACGACGGTCTAA	
		Probe (JOE/TAMRA)	CAAGCGTTGGATTGTTCACCCA	

*FAM*  6-carboxyfluorescein, *TAMRA*  6-carboxytetramethylrhodamine, *JOE*  4′,5′-dichloro-2′,7′-dimethoxy-6- carboxyfluorescein

### Preparation of mouse liver microsomes (MLµ)

Liver tissue (approx. 2.5 g) was minced and rinsed in ice-cold medium (10 mM HEPES, pH 7.4, 250 mM sucrose, 1 mM EDTA) to remove blood and bile. All following steps were performed on ice or at 4 °C. The tissue was homogenized in a Potter–Elvehjem homogenizer in medium containing 1 mg/ml fatty acid-free bovine serum albumin and 1 mM dithiothreitol. The homogenate was centrifuged at 800 × g for 10 min in a Sorvall RC-5B centrifuge (DuPont Instruments, Inula, Vienna, Austria) to remove nuclei and cell debris. The supernatant was centrifuged for 15 min at 11,000 × g to pellet mitochondria. To sediment MLµ, the mitochondria-free supernatant was centrifuged for 1 h at 100,000 × g in a Beckman LE-80 ultracentrifuge (Beckman Instruments, Vienna, Austria), and the pellet was dispersed in potassium phosphate buffer (100 mM, pH 7.4, 1 mM EDTA and 2 mM MgCl_2_). Protein content was determined by the Bradford method.

### Immunofluorescence microscopy

For immunofluorescence microscopy cryo-sections of liver tissue (4 µm thick, fixed in acetone at −20 °C for 10 min) were used as described previously [5]. Primary antibodies were monoclonal antibodies Ks 8.7, Ks 18.04 (Progen, Heidelberg, Germany), and 50K160 [5, 18] (detecting keratin 8 and 18), guinea pig anti-p62CT [3, 5], M_M_120-1 (detecting MDBs) [18, 19], chicken anti-CYP2A5/CYP2A6 (a kind gift of H. Raunio (Department of Pharmacology and Toxicology, University of Kuopio, Finland) [20], and rabbit anti-CYP2E1 (Chemicon International, Inc, Temecula, CA, USA). As secondary antibodies, Alexa Fluor 488 nm–conjugated goat anti-mouse IgG (Molecular Probes, Leiden, The Netherlands), Cy5™-conjugated goat anti-mouse Ig (Jackson Immune Research, West Grove, PA, USA), tetramethylrhodamine isothiocyanate (TRITC)-conjugated swine anti-rabbit Ig (Dako, Glostrup, Denmark), Rhodamine Red-X-conjugated goat anti-guinea pig IgG (Jackson), and FITC-conjugated rabbit anti-chicken IgG (Zymed, San Francisco, CA, USA) were used. Specimens were analysed with a Zeiss LSM 510 laser-scanning confocal microscope (Zeiss, Oberkochen, Germany). For control purposes, primary antibodies were omitted or replaced by isotype-matched Ig (Dako).

### CYP activities

For CYP activity assays frozen liver tissue was homogenized in Soerensen phosphate buffer (15 mM KH_2_PO_4_, 68 mM Na_2_HPO_4_ x H_2_O, pH 7.4) using an Ultraturrax T25 (Ika Labortechnik, Staufen, Germany). The homogenate (20% weight/volume) was centrifuged at 11,000 × g for 15 min. The supernatant was used in the assays [20–23]. Protein content was determined by the Bradford method. Coumarin 7-hydroxylase (COH) activity was determined with coumarin (100 µM) as substrate, the O-dealkylation of 7-ethoxyresorufin (EROD) activity was assessed by measuring fluorescence of resorufin using 1 µM ethoxyresorufin as substrate and the 7-ethoxycoumarin O-deethylation (ECOD) activity by measuring the fluorescence of hydroxycoumarin using 100 µM ethoxycoumarin as substrate. CYP activities were determined spectrophotometrically using a Hitachi fluorescence spectrophotometer F-2500 (Inula, Vienna, Austria) based on emission and excitation wavelengths of 390 nm and 450 nm (for COH and ECOD activities) and 530 nm and 580 nm (for EROD activity). Samples were assayed as duplicates.

### H_2_O_2_ formation by MLµ, CYP2A6 and CYP2E1 supersomes and its inhibition by bilirubin

H_2_O_2_ formation was measured using lucigenin (Invitrogen, Lofer, Austria) as chemiluminescence enhancer in the presence of an appropriate reducing system (P450 oxidoreductase from mouse liver cytosol, i.e., the supernatant after pelleting microsomes by ultracentrifugation) [24–26] in an Anthos-Labtec Lucy 1 (Anthos Labtec, Salzburg, Austria) luminometer using a white 96-cell microplate. In the assay MLµ (10 µg microsomal protein) or CYP2E1 and CYP2A6 supersomes equivalent to 250 fmol enzyme were dispersed in Soerensen buffer in the presence of 5 µM lucigenin. The reaction was started by adding 250 µM NADPH (total volume per well was 250 µl) and time-courses of the reactions were monitored for 1–2 h at 37 °C. In the inhibition experiments bilirubin (mixed isomers, Sigma-Aldrich, Vienna, Austria) was dissolved in 0.1 M KOH, diluted in Soerensen buffer and neutralized with 1 M HCl to give a 200 µM stock solution, which was stable on ice for 1 day. Alternatively, a 2 mM stock solution was prepared in the same way and stored at 20 °C.

### Statistical evaluation

For statistical evaluation SigmaPlot 15.0 (Inpixon, Palo Alto, CA, USA) was used. Significance levels were calculated by 1-way ANOVA all pairwise multiple comparison (Holm-Sidak).

## Results

### Determination of mRNAs of CYP2A5, Hmox-1 and p62 in mouse liver and COH activity at different stages of DDC intoxication in relation to MDB formation (Fig. 1A(a-e), B)

**Fig. 1:**
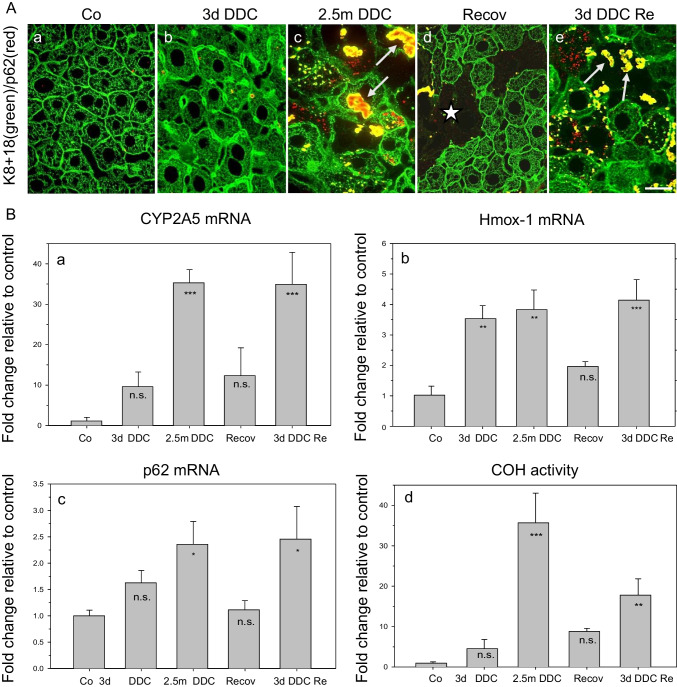
**A** Double-label immunofluorescence microscopy using antibodies to keratin K8 + 18 (green) and p62 (red). **a** Control liver (Co): hepatocytes display regular keratin IF cytoskeleton. **b** DDC feeding for 3 days (3d DDC): hepatocytes are enlarged with anisokaryosis but intact keratin IF cytoskeleton, which is more concentrated around bile canaliculi **c** DDC feeding for 2.5 months (2.5 m DDC): variably sized, mostly enlarged hepatocytes, some with preserved and some with diminished or missing keratin IF cytoskeleton; appearance of larger compact and smaller granular MDBs co-expressing keratin and p62 (arrows). **d** 1 month recovery on standard diet after DDC feeding for 2.5 months (Recov): areas of hepatocytes without visible keratin IF cytoskeleton (“empty cells”, asterisk) and disappearance of most MDBs in addition to hepatocytes with preserved keratin IF cytoskeleton. **e** DDC refeeding for 3 days of mice DDC-fed for 2.5 months and recovered on standard diet for one month (3d DDC Re): reappearance of compact and granular MDBs in enlarged hepatocytes lacking keratin IF cytoskeleton (arrows). Bar, 30 µm. **B** mRNAs of CYP2A5, Hmox-1, p62 and CYP2A5-dependent coumarin-7-hydroxylation (COH) as fold change relative to control. **a** DDC feeding for 2.5 months leads to a significant increase of CYP2A5 mRNA expression *(*2.5 m DDC) compared to controls (Co) and mice fed DDC for 3 days (3d DDC), whereas in the recovery period (Recov) the values decrease to values not significantly different from controls. CYP2A5 mRNA expression is again significantly increased after refeeding for 3 days (3d DDC Re); CYP2A5 mRNA of 3 d DDC Re mice is significantly higher than that of 3 d DDC mice (3d DDC Re versus 3 d DDC, *p* < 0.01). **b** Hmox-1 mRNA expression in mouse liver is significantly increased already by DDC feeding for 3 days (3d DDC) and 2.5 months (2.5mo DDC) in comparison to controls (Co) and decreased in the recovery period. Refeeding for 3 days results again in significant elevation; no significant difference was observed between 3 d DDC Re and 3 d DDC. **c** p62 mRNA is elevated (not significantly in comparison to controls) already after 3 days of DDC feeding (3d DDC), increased further in 2.5 months-fed mice (2.5 m DDC), decreased in the recovery period (Recov) and increased again after refeeding for 3 days (3d DDC Re; n.s., not significant). A significant difference exists between 3 d DDC Re and 3 d DDC (*p* < 0.05). **d** Coumarin-7-hydroxylation (COH) in mouse liver at different stages of DDC feeding, recovery and re-feeding. Enzyme activity is significantly increased in 2.5 months DDC-fed mouse liver (2.5 m DDC) in comparison to controls (Co) and mice fed DDC for 3 days (3d DDC), diminished in the recovery period (Recov) and increased significantly again after DDC refeeding. The difference between 3 d DDC Re and 3 d DDC is significant (*p* < 0.01). Bars show values (mean ± standard deviation) calculated as fold change compared to the expression in control mice (significances as compared to control **a**–**d**: ***p < 0.001; **p < 0.01; *p < 0.05; n.s., not significant)

Livers of mice at 3 days and 2.5 months of DDC intoxication, after 1 month recovery and at 3 days of re-intoxication were evaluated histologically and by double-label immunofluorescence microscopy for MDB formation. The morphologic results corresponded to those previously described [1, 4, 5]. After 3 days of DDC feeding, no MDBs were present, and the expression of CYP2A5 mRNA did not significantly differ from that of untreated control mice. However, Hmox-1 expression was already significantly elevated at this stage. After 2.5 months of DDC intoxication numerous MDBs were present and CYP2A5 as well as Hmox-1 mRNAs were overexpressed. After the recovery period of 1 month, MDB-containing hepatocytes were highly reduced. After 3 days of DDC re-intoxication of recovered (primed) mice numerous MDBs re-appeared accompanied by overexpression of CYP2A5 and Hmox-1 mRNAs. P62 mRNA behaved similarly. DDC-dependent overexpression of CYP2A5 mRNA mirrored the occurrence of MDBs, which was also reflected by significantly increased (predominantly) CYP2A5-dependent COH activity (Fig. 1B). In contrast, ECOD and EROD activities, related to other cytochromes, were much lower at the different stages of DDC treatment and without association with MDB occurrence except EROD activity, which was elevated (although to a much lower degree than COH) only in mice fed DDC for 2.5 months but not after refeeding for 3 days (suppl. Figure 1).

### Association of CYP2A5 expression with presence of MDBs at the cellular level

**Fig. 2:**
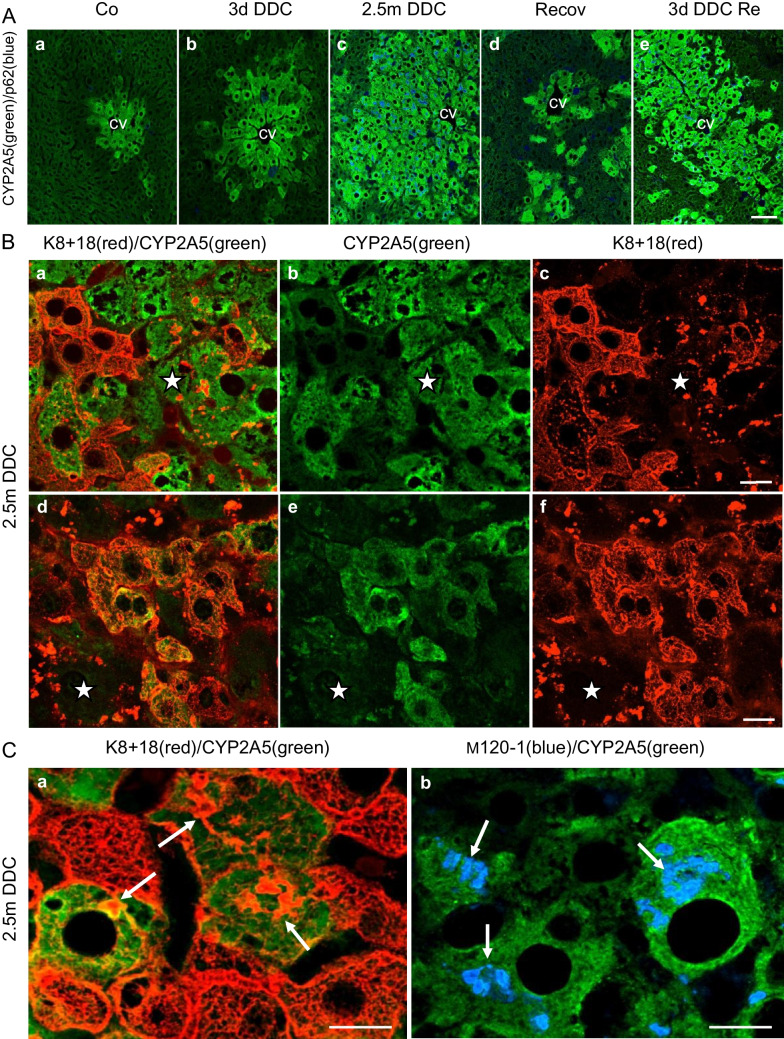
Double-label immunofluorescence microscopy. **A** Hepatocytic CYP2A5 expression (green) in control mice, mice fed DDC for 3 days, mice fed DDC for 2.5 months, recovered mice and recovered mice refed DDC for 3 days. **a** In control mice (Co) CYP2A5-positive hepatocytes are present in centrilobular position. **b** In 3 days DDC-fed mice (3d DDC) the number of CYP2A5-positive hepatocytes in centrilobular position is increased in comparison to controls. **c** After 2.5 months of DDC feeding (2.5 m DDC) CYP2A5-positive hepatocytes occupy almost the whole lobule, whereas in the recovery period (Recov) the number of CYP2A5-positive hepatocytes is markedly reduced (**d**), but increased again after 3 days of DDC re-intoxication (3d DDC Re; **e**). MDBs are immunostained with the antibody to p62 (blue). cv, central vein; Bar, 100 µm. **B** double-label immunofluorescence microscopy of CYP2A5/CYP2E1 (green) and keratin K8/18 (red) expression in hepatocytes of mice fed DDC for 2.5 months (**a**–**c**). Hepatocytes with lack of keratin IF immunostaining (“empty cells”) containing MDBs are positive for CYP2A5 (asterisk). CYP2A5 is also present in hepatocytes with preserved cytoskeleton. In contrast, antibodies to CYP2E1 (**d**–**f, asterisk**) revealed immunostaining of hepatocytes with preserved keratin IF cytoskeleton but not of hepatocytes lacking keratin IF cytoskeleton and MDB formation. Bar, $30 \mu \mathrm{m}$. **C** Higher magnification related to **B(a–c)**. **a** CYP2A5 is expressed in enlarged hepatocytes with reduced or lacking keratin IF cytoskeleton containing keratin-positive MDBs (arrows); **b** MDBs (blue color) are specifically visualized using the MDB-specific M_M_ 120–1 antibody (arrows). Bar, 40 µm

Because of the striking elevation of CYP2A5 mRNA and COH activity in mice in relation to the appearance of MDBs, we focused on in situ detection of this cytochrome in hepatocytes. In normal control livers CYP2A5 was only demonstrable by immunofluorescence in the centrilobular region. The CYP2A5-positive area extended with duration of DDC feeding. After 2.5 months of DDC feeding, CYP2A5-positive hepatocytes occupied almost the whole lobule. In the recovery period the number of CYP2A5-positive hepatocytes was reduced and concentrated again in the centrilobular region, but increased within 3 days of DDC re-intoxication (Fig. 2A(a-e)). Furthermore, CYP2A5 protein correlated with the presence of MDBs at the cellular level; double-label immunofluorescence microscopy using antibodies against K8/K18 and CYP2A5 demonstrated that hepatocytes with disrupted keratin intermediate filament (IF) network (ballooned cells) and cells containing MDBs were strongly positive for CYP2A5 (Fig. 2B(a–c); 2C(a, b)). In contrast, CYP2E1-related immunostaining did not show an association with IF cytoskeleton alterations and MDB formation (Fig. 2B(d-f)).

### Production of H_2_O_2_ by CYP2A5 by MLµ and its inhibition by bilirubin

**Fig. 3:**
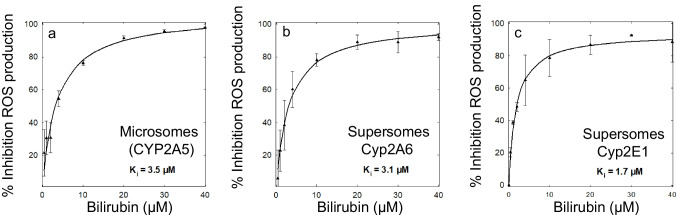
Inhibition of CYP-derived ROS production by mouse liver microsomes and CYP2A6-and 2E1-containing human supersomes by bilirubin. **a** Mouse liver microsomes (10 µg/well); **b** CYP2A6 supersomes (250 fmol/well) and **c** CYP2E1 supersomes (250 fmol/well) were incubated with 250 µM NADPH, 5 µM lucigenin, and the indicated concentrations of bilirubin

MLµ incubated with NADPH and lucigenin as reporter showed H_2_O_2_ formation (indicating oxidative stress) (Fig. 3). This is consistent with the observation that CYPs as leaky cytochromes preferentially produce H_2_O_2_ [27, 28]. Bilirubin strongly inhibited CYP2A5-mediated H_2_O_2_ production by MLµ (apparent K_i_ = 3.5 µM; Fig. 3a). H_2_O_2_ formation was also inhibited by coumarin and DDC, other substrates of CYP2A5, although this effect was less pronounced (apparent K_i,_ 157 µM and 50 µM, respectively; not shown). Moreover, with supersomes containing its human orthologue, CYP2A6, and with CYP2E1-containing supersomes a comparable inhibition of H_2_O_2_ production by bilirubin (apparent K_i_ = 3.1 µM and apparent K_i_ = 1.7 µM, respectively) was observed (Fig. 3b, c).

## Discussion

The chemical composition of MDBs in mice and humans consisting of stress-related proteins (i.e., keratins, heat shock proteins, p62) supports their relationship to chronic cellular stress [1–5]. Our results demonstrate the importance of CYP2A5 in the interplay of oxidative stress and related oxidative and antioxidative pathways in association with MDB formation in the DDC-intoxicated animals; they underline similarities in the role of CYPs between chronic DDC intoxication of mice and steatohepatitis of humans and support findings by other authors regarding the role of xenobiotic metabolism and oxidative stress in MDB formation [8, 10–17]. CYP2E1, CYP2A6, and its murine orthologue CYP2A5 are regarded as “leaky” since they spontaneously produce reactive oxygen species, particularly H_2_O_2_, in the presence of NADPH or NADH [30, 31].

Bilirubin is the product of Hmox-1- dependent heme catabolism and an excellent substrate of CYP2A5 preferentially resulting in biliverdin [29–34]. The antioxidative effect of bilirubin is amplified by the cyclic conversion of bilirubin and biliverdin [35–38]. It is noteworthy in this context that bilirubin and glutathione have complementary antioxidative effects, since glutathione primarily protects soluble proteins whereas the bilirubin-biliverdin cycle is preferentially responsible for lipid protection, although water-soluble biliverdin may also be effective on water-soluble targets [36].

It has been shown that bilirubin treatment of primary hepatocytes increased CYP2A5 protein without affecting mRNA levels, i.e., by stabilization of the protein in its active conformation [31, 32, 37]. CYP2A5 is also upregulated by various xenobiotics whenever total CYP content decreases and Hmox-1 activity increases suggesting compensatory feedback mechanisms to maintain the CYP2A5 activity in the state of intracellular heme level reduction [30, 31, 39, 40]. Coordinated regulation of Hmox-1 and CYP2A5 expression is crucial for the balance between bilirubin production and degradation. The induction of Hmox-1 promotes heme degradation, which leads to depletion of the cellular heme pool as a possible reason for reduced general cellular CYP content. Therefore, the elevation of CYP2A5 mRNA and CYP2A5-related catalytic activity (with coumarin as high affinity substrate of CYP2A5) observed in our studies with DDC-intoxicated mice, suggest the preferential incorporation of heme into CYP2A5 under these conditions. Our results agree with Abu-Bakar et al. [31–33, 39] that CYP2A5 may be upregulated in pathologic conditions associated with compromised functions of most other CYPS and suppression of total hepatic CYP expression. The ability of bilirubin to efficiently inhibit CYP2A5-induced H_2_O_2_ formation provides feedback limitation to damage by ROS [31, 32, 34–37].

The observation that DDC results in a cascade of the adaptive responses of the cell to oxidative stress is underlined by the previously shown activation of the transcription factor Nrf2 in DDC-fed mice (41), which regulates the coordinated expression of genes encoding a variety of antioxidant enzymes, such as Hmox-1, biliverdin reductase and glutathione S-transferases [42, 43]. Furthermore, p62 a constant component of MDBs, is a binding partner of Keap1 and prevents Keap1 from trapping Nrf2 leading to Nrf2 stabilization and activation [41, 43] underlining a possible functional link to MDB formation.

**Fig. 4:**
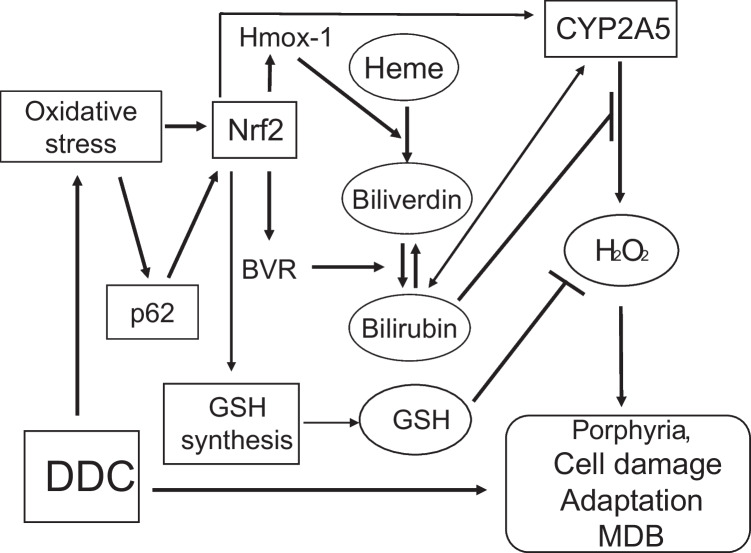
Schematic summary of suggested interplay of oxidative stress, heme metabolism and antioxidative pathways finally resulting in porphyria, protein aggregation, adaptation and MDB formation in DDC intoxicated mouse livers

The goal of our studies was to uncover similarities between the DDC mouse model and human steatohepatitis with emphasis on MDB formation (Fig. 4). We demonstrate the important role of oxidative stress associated with increased CYP2A5 and identify the bilirubin-biliverdin axis as potent endogenous inhibitor of H_2_O_2_ production, Bilirubin is cytotoxic at high concentrations, but a potent cytoprotective antioxidant at lower physiologic concentrations. The similarities between the effects of CYP2A5 in the DDC mouse model and CYP2A6 and CYP2E1 in steatohepatitis of humans suggest that MDB formation results from similar pathophysiological mechanisms. Although animal models are indispensable tools to elucidate pathogenic principles of human disease, no single experimental model reproduces all pathogenic aspects of human disease [4]. However, reproduction and analysis of highly characteristic morphologic features, like MDBs, may allow identification of common pathophysiological mechanisms even in disorders of different etiology.

## Supplementary Information

Below is the link to the electronic supplementary material.

Supplementary Material 1 Suppl. Fig.1: EROD and ECOD activities are very low as compared with COH activity (Fig. 1, B, d; note the different scale on the ordinate) in the different stages of DDC treatment and recovery and not significantly different from the untreated control with the exception of EROD activity in mice DDC- intoxicated for 2.5 month (2.5 mo DDC), which was elevated in comparison to control (Co) (** p < 0.01; n.s., not significant). (PPTX 46.8 MB)

## Data Availability

The paper contains all data of the study.

## Abbreviations

ASH: Alcohol-related steatohepatitis
COH: Coumarin hydroxylase
CYP: Cytochrome P450
DDC: 3,5-Diethoxycarbonyl-1,4-dihydrocollidine
ER: Endoplasmic reticulum
Hmox-1: Heme oxygenase-1
IF: Intermediate filament
MDB: Mallory-Denk body
MASH: Metabolic dysfunction-associated steatohepatitis
MLµ: Mouse liver microsomes
PP-IX: Protoporphyrin IX
ROS: Reactive oxygen species
