# Supplementary material for: Cytochrome P450 as possible link between Mallory-Denk body formation in DDC-intoxicated mouse liver and human steatohepatitis
Source: Virchows Arch. 2025 Oct 30;488(6):1265–74. doi: 10.1007/s00428-025-04290-4 (PMC13264601; doi:10.1007/s00428-025-04290-4)
Supplement: Supplementary file 1 — Supplementary Material 1 Suppl. Fig.1: EROD and ECOD activities are very low as compared with COH activity (Fig. 1, B, d; note the different scale on the ordinate) in the different stages of DDC treatment and recovery and not significantly different from the untreated control with the exception of EROD activity in mice DDC- intoxicated for 2.5 month (2.5 mo DDC), which was elevated in comparison to control (Co) (** p < 0.01; n.s., not significant). (PPTX 46.8 MB) [file 428_2025_4290_MOESM1_ESM.pptx]

## Slide 1
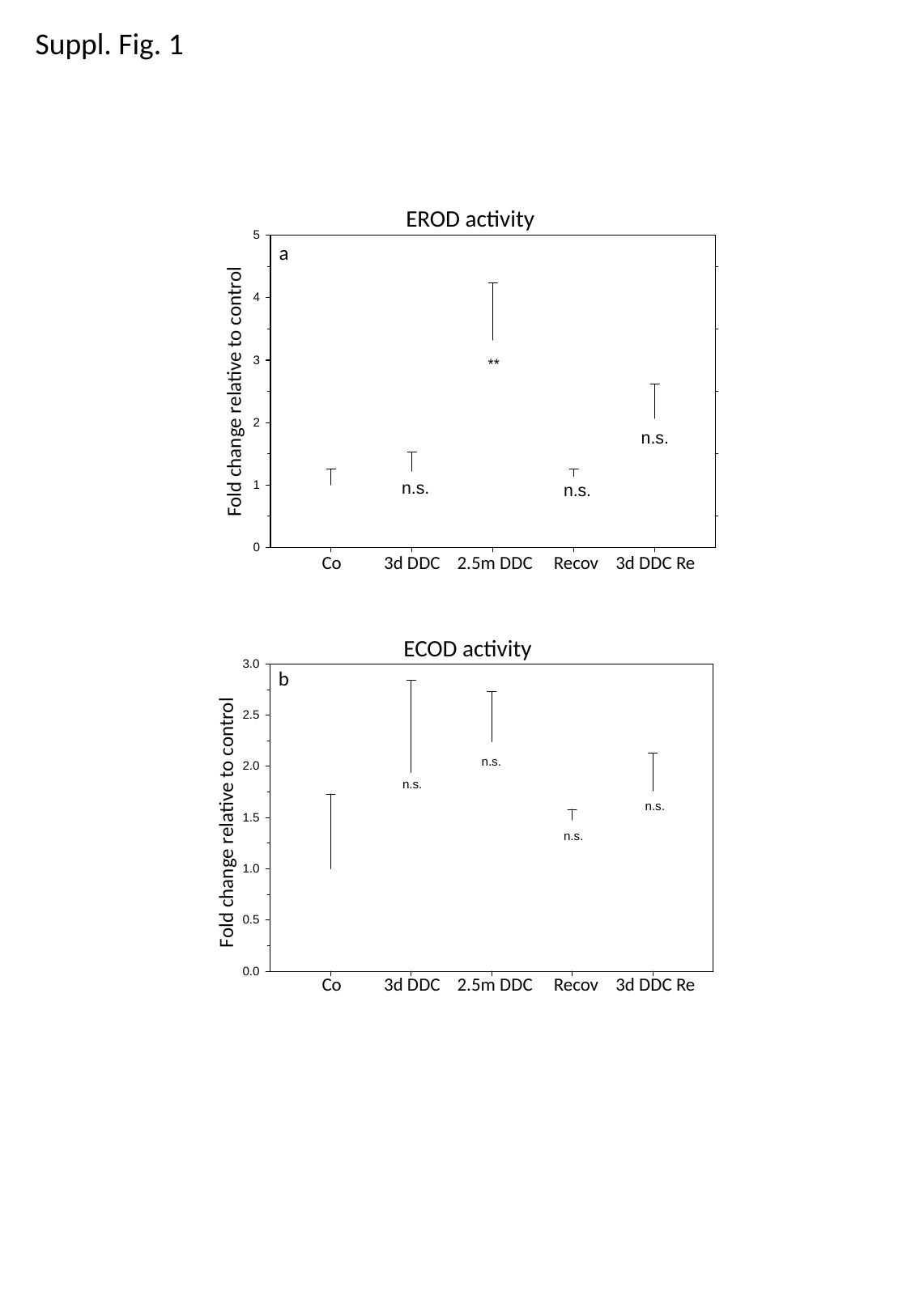

COH
Suppl. Fig. 1
EROD activity
a
Fold change relative to control
 Co 3d DDC 2.5m DDC Recov 3d DDC Re
ECOD activity
b
Fold change relative to control
 Co 3d DDC 2.5m DDC Recov 3d DDC Re
